# Emphasizing responder speed or accuracy modulates but does not abolish the distractor-induced quitting effect in visual search

**DOI:** 10.1186/s41235-023-00516-8

**Published:** 2023-10-10

**Authors:** Rebecca K. Lawrence, B. A. Cochrane, A. Eidels, Z. Howard, L. Lui, J. Pratt

**Affiliations:** 1 Griffith University, Southport, Australia; 2 University of Aberdeen, Aberdeen, UK; 3 The University of Newcastle, Newcastle, Australia; 4 University of Western Australia, Crawley, Australia; 5 University of Toronto, Toronto, Canada

**Keywords:** Quitting thresholds, Visual search, Distraction

## Abstract

When a highly salient distractor is present in a search array, it speeds target absent visual search and increases errors during target present visual search, suggesting lowered quitting thresholds (Moher in Psychol Sci 31(1):31–42, 2020). Missing a critical target in the presence of a highly salient distractor can have dire consequences in real-world search tasks where accurate target detection is crucial, such as baggage screening. As such, the current study examined whether emphasizing either accuracy or speed would eliminate the distractor-generated quitting threshold effect (QTE). Three blocks of a target detection search task which included a highly salient distractor on half of all trials were used. In one block, participants received no instructions or feedback regarding performance. In the remaining two blocks, they received instructions and trial-by-trial feedback that either emphasized response speed or response accuracy. Overall, the distractor lowered quitting thresholds, regardless of whether response speed or response accuracy was emphasized in a block of trials. However, the effect of the distractor on target misses was smaller when accuracy was emphasized. It, therefore, appears that while the distractor QTE is not easily eradicated by explicit instructions and feedback, it can be shifted. As such, future research should examine the applicability of these and similar strategies in real-world search scenarios.

**Supplementary Information:**

The online version contains supplementary material available at 10.1186/s41235-023-00516-8.

## Introduction

Efficiently searching for objects of interest is crucial for day-to-day activities such as wayfinding and shopping, as well as professions such as radiology and baggage screening (Biggs and Mitroff, 2015; Biggs et al., 2013; Drew et al., 2013; Ganesan et al., 2018). Consequently, a large body of theoretical and experimental work evaluating the impact of individual and task-based factors on search processes has accumulated over the past 30 years, with one of the most studied areas being visual distraction (Biggs et al., 2017; Boot et al., 2005; Burnham, 2010; Burnham et al., 2014; Duncan and Humphreys, 1989; Egeth et al., 1984; Grubert et al., 2013; McCarley, 2009; Moher, 2020; Paoletti et al., 2015; Peltier and Becker, 2017; Theeuwes, 1992; Treisman and Gelade, 1980; Treisman and Sato, 1990; Wolfe, 2021; Wolfe et al., 1989). However, research exploring the effects of salient visual distractors on performance in search tasks where a target is either present or absent is minimal (Moher, 2020). Such research is critical as in many real-world search tasks which contain distractors, the target is sometimes (or even often) absent (Moher, 2020; Wolfe et al., 2007).

**Fig. 1:**
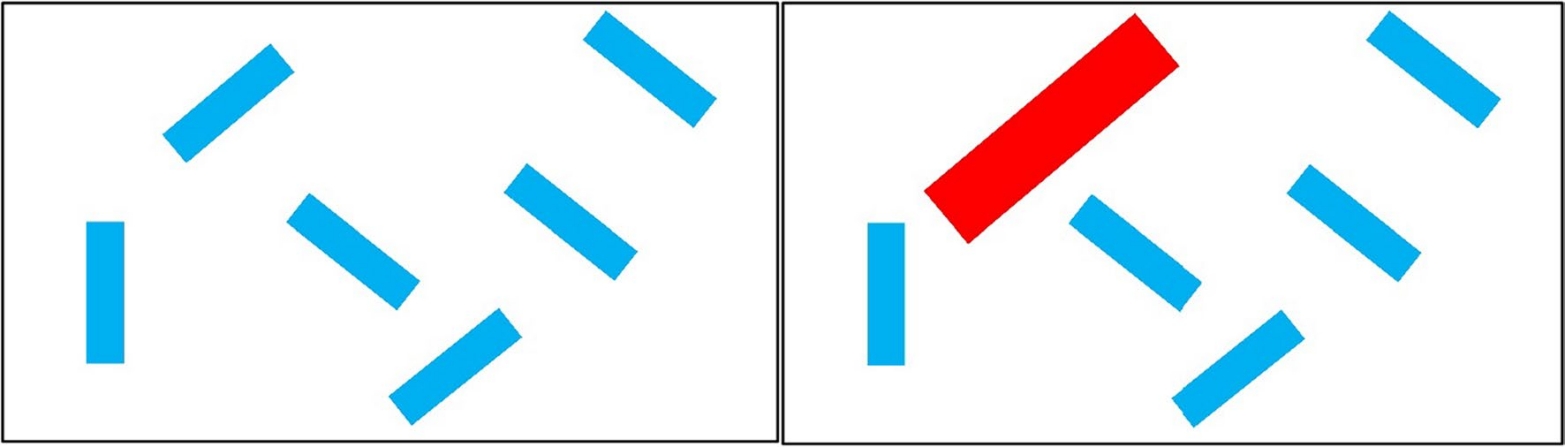
Example of a target detection visual search task in the absence (**a**) or presence (**b**) of a highly salient distractor based on Moher (2020)

To address this gap in the literature, Moher (2020) used a search task including a target rectangle that was vertically oriented and present on either 50% (Experiment 1) or 20% (Experiment 2) of trials among slanted filler rectangles of the same color (Fig. 1a). Further, on half of the trials, one of the filler rectangles was replaced by a much larger, differently colored, delayed onset distractor (Fig. 1b; note we use the term “distractor” to refer to this salient item and “filler” for the standard non-target items). Response times and error rates were then examined as a function of distractor and target presence. It was found that while distractors resulted in slower response times on target present trials, they caused faster response times on target absent trials. Furthermore, although error rates were largely unaffected by the distractor on target absent trials, more errors were made when a distractor was present compared to absent on target present trials. As such, the findings suggested that the salient distractors influenced how long individuals searched before deciding a target was absent, suggesting a lower search quitting threshold, or a distractor quitting threshold effect (QTE; Chun and Wolfe, 1996; Lawrence and Pratt, 2022; Moher, 2020; Moran et al., 2013; Wolfe, 2012, 2021; Wolfe and Van Wert, 2010).

To date, research has not converged on a unified mechanism to explain the distractor QTE; however, some possibilities have been explored (Lawrence and Pratt, 2022; Lawrence et al., 2023a; Lui et al., 2023; Moher, 2020). For example, Lawrence and Pratt (2022) found that the size of the distractor was important, with the distractor QTE only emerging when the distractor was larger than the other array items (compared to the same size as the other items). Furthermore, in the original QTE study, Moher (2020) found that the magnitude of the quitting effect was smaller when target prevalence was 20% compared to 50%. Finally, while Lawrence et al. (2023a) found that the timing of the appearance of the distractor had little effect on the QTE, Lui et al. (2023) observed an effect of distractor prevalence. Specifically, in the second experiment of this study, participants completed the standard target detection search task when the distractor was present on 90% of trials or 10% of trials. Interestingly, the distractor QTE emerged only in the high distractor prevalence condition. In the 10% prevalence condition, although the distractors increased target misses, they did not modulate response speeds. One reason that this might have occurred is because the salience of the low prevalence distractor was so high that the attentional capture generated by it mitigated the distractor-based speeding effect (Lui et al., 2023). Nonetheless, it appears that overall, the distractor QTE is most likely to emerge when very distinctive distractors are processed in a way that maximizes search efficiency (perhaps at the cost of accuracy).

More generally, the distractor QTE may be problematic in searches where accurate target identification is crucial, as the distractor makes it more likely for an observer to miss a target when present (Moher, 2020). For example, consider a security screener at the airport searching X-rays of carry-on baggage. One bag may contain both a threat (e.g., small box cutters) and a highly salient distractor (e.g., a large laptop) among smaller distracting filler items (e.g., tissues, headphones, makeup, and pen). Based on the distractor QTE, it is more likely that one might miss the box cutters when the salient distracting item is also present as the search is more quickly stopped. Although the visual complexity of tasks used to study the QTE may not exactly reflect the constraints of real-world search like baggage screening, it is important to note that simple, laboratory-based visual search experiments can be effectively used to explore the cognitive mechanisms underlying accurate search performance in applied settings. Indeed, Biggs et al. (2013) were able to differentiate performance across a group of the USA Transport Security Authority baggage screeners (including both early career and experienced screeners) and a non-expert university sample when completing a simple visual search task which used shape stimuli on a blank background. As such, it is essential to explore strategies which could potentially alleviate the distractor generated lowering of quitting thresholds due to its potential negative impacts on real-world search performance.

One potential strategy which could be used to modulate the distractor QTE is task instructions (e.g., Cox et al., 2021; Heitz, 2014; and McCarley, 2009). For example, in a laboratory-based baggage screening search task that did not contain salient distractors, McCarley (2009) found that encouraging fast compared to accurate responding influenced search quitting thresholds. At the beginning of a series of trials, participants were shown instructions stating that they should “Emphasize ACCURACY” or “Emphasize SPEED” and were asked to search for knives (included on 40% of trials) in X-ray images of a bags typically seen at the airport. Compared to when accuracy was encouraged, encouraging speed decreased accuracy when the target was present (no differences were found when the target was absent), and reaction times were faster with the difference being most pronounced for the target absent trials (note that eye movements were also recorded but the findings are not discussed here). Furthermore, Cox et al. (2021) recently found that instructions given to participants before completing a search task containing no performance feedback significantly framed perceptions of target frequency and thus influenced quitting thresholds. In the study, participants searched an array of 25 objects for 1 or 2 target shapes. Prior to completing the search task, half of the participants were informed that on each trial, there were 0, 1, or 2 targets to be found. In contrast, the other half of participants were told that there were 1 or 2 targets to be found. As such, one group anticipated target absent trials throughout the study whereas the other group did not. Importantly, despite there always being at least one target present on every trial, on trials where only one target was present, those who expected target absent trials tended to miss the target more often than those who always expected a target, as well as end searches earlier. Finally, authors such as Forstmann et al. (2011) have used computational models of decision-making to show that speed vs accuracy instructions primarily influence decision thresholds, such that speed emphasis reduces the amount of evidence required to execute a response. This aligns well with the quitting threshold explanation of the distractor effect.

As well as initial task instructions, other strategies which could be used to alter the distractor QTE include trial-by-trial feedback (“Correct!” “Incorrect!” and “Too Slow!”), time penalties for incorrect or slow responding, and the use of points-based systems (e.g., Fitts, 1966; Heitz, 2014; Navalpakkam et al., 2009; Schwark et al., 2012; and Wolfe et al., 2007). Indeed, these and similar strategies have been found to shape quitting thresholds in the context of the low prevalence effect (LPE), where observers terminate search earlier for low target prevalence search tasks (e.g., Wolfe et al., 2005). For example, Schwark et al. (2012) found that they could minimize the LPE in a visual search task by telling participants that they missed a target, when, in reality, it was a target absent trial. Specifically, for the low prevalence blocks of the experiment, providing participants with inaccurate feedback increased the number of false alarms and decreased the number of misses on target present trials compared to when accurate feedback was provided. Furthermore, Wolfe et al. (2007) found that adding high prevalence blocks of trials with correct accuracy feedback into a search task containing low prevalence blocks of trials with no feedback removed the LPE (Experiment 7). However, it is also worth noting that the authors reported that “in pilot experiments, changing payoff matrices did not have a noticeable effect on miss error rates” (Wolfe et al., 2007, p. 3), and that feedback intended to slow target absent responses did not remove the LPE (Experiment 2). Thus, the effect of trial-by-trial feedback on search quitting thresholds may be somewhat nuanced.

Nonetheless, given that both task instructions and trial-by-trial feedback appear to be able to influence performance during visual search tasks, and that these performance changes likely impact decision thresholds, it is reasonable to expect that similar strategies will be able to modulate the distractor QTE. Given this possibility, we adapted the original search task developed by Moher (2020) to directly test whether instructions and feedback emphasizing either speed or accuracy would modulate the phenomenon. Specifically, participants completed three blocks of experimental trials. In the first block, no feedback regarding speed or accuracy performance was provided. In the remaining two blocks (order randomized across participants), the task was completed with instructions and trial-by-trial feedback to encourage either fast or accurate responding. Overall, it was expected that the distractor QTE may be mitigated for the accuracy emphasized compared to the speed emphasized condition. That is, when participants were asked to respond as accurately as possible, they may be less likely to prematurely terminate search in the presence of a salient distractor.

Despite explicit trial-by-trial feedback being difficult to implement in some applied settings (e.g., one cannot provide correct/incorrect feedback on every bag screened at the airport; see Cox et al., 2021), testing some of the potential ways in which one could remove or modulate the QTE is a reasonable approach for exploring the mechanisms underlying the phenomenon. Indeed, if the QTE emerges due a shift in search strategy, this will provide important information on how top–down goals can help avoid the QTE or, at least, mitigate its effect. Furthermore, even if true corrective feedback cannot be used in applied settings, feedback during training sessions alongside instructions emphasizing accuracy could be implemented (e.g., McCarley, 2009; Schwark et al., 2012; and Wolfe et al., 2007). Thus, the current study is an important first step in developing solutions to the distractor QTE.

## Method

### Participants

Two-hundred and four individuals completed the online study. Two participants who self-reported vision problems were excluded from analyses. However, seven participants who did not report any demographic information and one participant who reported all demographic information except for their vision status were retained. For the participants in the final sample who reported demographic information (*N* = 195; 140 = female, 51 = male, and 4 = other), the mean age was 24.48 years (*SD* = 6.83 years). One hundred and seventy-five reported that they were right-handed, 15 reported that they were left-handed, and five reported that they were ambidextrous. All participants were compensated with course credit and gave informed consent to participate.

### Stimuli and procedure

After providing informed consent and reporting demographic characteristics via LimeSurvey (Limesurvey, n.d.), participants completed an online visual search task like that used in recent research on distraction and quitting thresholds (e.g., Lawrence and Pratt, 2022 and Moher, 2020). PsychoPy and Pavlovia were used to create and run the study (Peirce et al., 2022), with participants using their own devices. Given that internet browsers, monitors, and operating systems of participants varied, the stimulus specifications used to program the study are reported.

On each trial, participants searched for a blue target rectangle that was vertically oriented (40 by 8 pixels) among filler rectangles that were oriented 30 degrees to the left or right from vertical. The target was present on 50% of trials. The array size was six shapes, and the shapes appeared where the lines of a 500 by 500-pixel grid (with 50 pixels spacing) converged. On one half of the trials, the filler rectangles were all the same size and color as the target. However, on the other half of trials, a large (80 by 16 pixels) red rectangle replaced one of the filler rectangles. All shapes appeared at the same time, and there was an intertrial interval of approximately 1.5 s. Participants responded target present by pressing z and target absent by pressing m on their keyboard. Trial type order was randomly intermixed for each participant with each combination of target and distractor presence occurring an equal number of times.

After completing eight practice trials with corrective feedback (i.e., “correct” or “press z for when there is a vertical line and ‘m’ when there is not”), there were three experimental blocks of trials: a baseline block, an accuracy block, and a speed block. The baseline block was always presented first and contained 208 trials where neither speed nor accuracy of response was emphasized in instructions, and no trial-by-trial feedback was provided. After this, participants completed 208 trials that emphasized accuracy (or speed), followed by 208 trials that emphasized speed (or accuracy). The order of the speed and accuracy emphasis blocks was randomly chosen for each participant with rest breaks offered every 104 trials.

In the accuracy emphasis block, participants were informed that it was very important they responded accurately. They were told that they would be awarded a point for each correct response and informed of the maximum total number of points for a series of trials they could achieve (a maximum of 104 points could be awarded for each sequence of 104 trials which reflects the number of trials between interspersed rest breaks). They were also told that they would receive feedback if they responded inaccurately. For each correct response, participants were shown a “Correct!” message for 1 s and were informed of the total number of points that they had accumulated. For an incorrect response, a message saying “Incorrect! Please respond accurately!” appeared for 5 s, and no information about total points accrued was provided.

In the speed emphasis block, participants were informed that it was very important they responded as quickly as possible. They were informed that they would be awarded points for fast responses and receive feedback messages for slow responses. They were also informed of the total number of points they could achieve for a series of trials. Fast responses were classified as those that were faster than participants trimmed mean reaction time (RT) from the baseline block (responses < 200 ms or > 3 s were removed from the calculation^1^). Thus, during this block, search stimuli only remained on screen for the duration of the participants mean RT. If participants responded quickly (regardless of accuracy), a “Fast Response!” feedback message was displayed for 1 s as well as their total points accrued. However, if participants did not respond in time, a feedback message “Too slow! Please respond quickly!” was displayed for 5 s with no points information shown. Thus, there was an incentive for participants to respond accurately or quickly in the corresponding block. Overall, the experiment took around 40 min to complete.

## Results

### Data screening and cleaning

Prior to our main analyses, 11 participants were excluded because either their overall accuracy in the experiment was 60% or lower, their accuracy was 60% or lower in the accuracy instruction block, or if any condition (i.e., target presence, distractor presence, and instruction [baseline, speed emphasis, and accuracy emphasis] cell) had an accuracy of 10% or lower (see Lawrence and Pratt, 2022 for a similar method). Furthermore, correct RT data were examined at the individual level. Responses faster than 200 ms or slower than 10 s were trimmed when calculating correct mean RTs for each condition (The mean number of correct trials that were excluded per participant in the final sample was 0.93%). For the remaining 191 participants, outliers for accuracy, followed by correct mean RT outliers, were removed at the group level. Twenty-five participants whose accuracy or correct mean RT exceeded ± 3.29 standard deviations in any condition of the experiment [baseline, speed emphasis, and accuracy emphasis by target and distractor presence] were removed leading to a final sample of 166 participants (this outlier cutoff corresponds to 0.1% data points per condition per variable; Tabachnick and Fidell, 2013).^2^

### The distractor QTE

The distractor QTE is studied by examining target absent correct mean RTs and target present accuracy as a function of distractor presence (Moher, 2020). As such, we conducted two separate ANOVAs on the correct mean RT and accuracy data for these key conditions to examine the effect of speed versus accuracy instructions/feedback on the QTE. Descriptive data for all conditions are found in Tables 1 and 2. Given that participants always completed the baseline block first, direct comparisons were made only between the speed and accuracy emphasis blocks for our main analyses. Nonetheless, an analysis of the baseline data showing the presence of a quitting effect can be found in the Additional file 1 for this paper.

**Table 1:** Mean correct RT (standard deviation) as a function of block, distractor presence, and target presence

	Distractor present	Distractor absent
Target present		
Baseline	1127 ms (279 ms)	1072 ms (252 ms)
Accuracy emphasized	1003 ms (275 ms)	969 ms (263 ms)
Speed emphasized	695 ms (147 ms)	679 ms (141 ms)
Target absent		
Baseline	1433 ms (414 ms)	1493 ms (436 ms)
Accuracy emphasized	1361 ms (442 ms)	1424 ms (480 ms)
Speed emphasized	780 ms (190 ms)	794 ms (197 ms)

**Table 2:** Mean accuracy (standard deviation) as a function of block, distractor presence, and target presence

	Distractor present	Distractor absent
Target present		
Baseline	88.06% (9.40%)	91.31% (7.73%)
Accuracy emphasized	95.27% (4.53%)	96.90% (3.42%)
Speed emphasized	74.61% (16.15%)	78.35% (15.09%)
Target absent		
Baseline	98.76% (2.45%)	99.02% (1.95%)
Accuracy emphasized	99.59% (0.92%)	99.49% (1.16%)
Speed emphasized	87.19% (12.18%)	85.30% (12.55%)

#### RT data for target absent trials

**Fig. 2:**
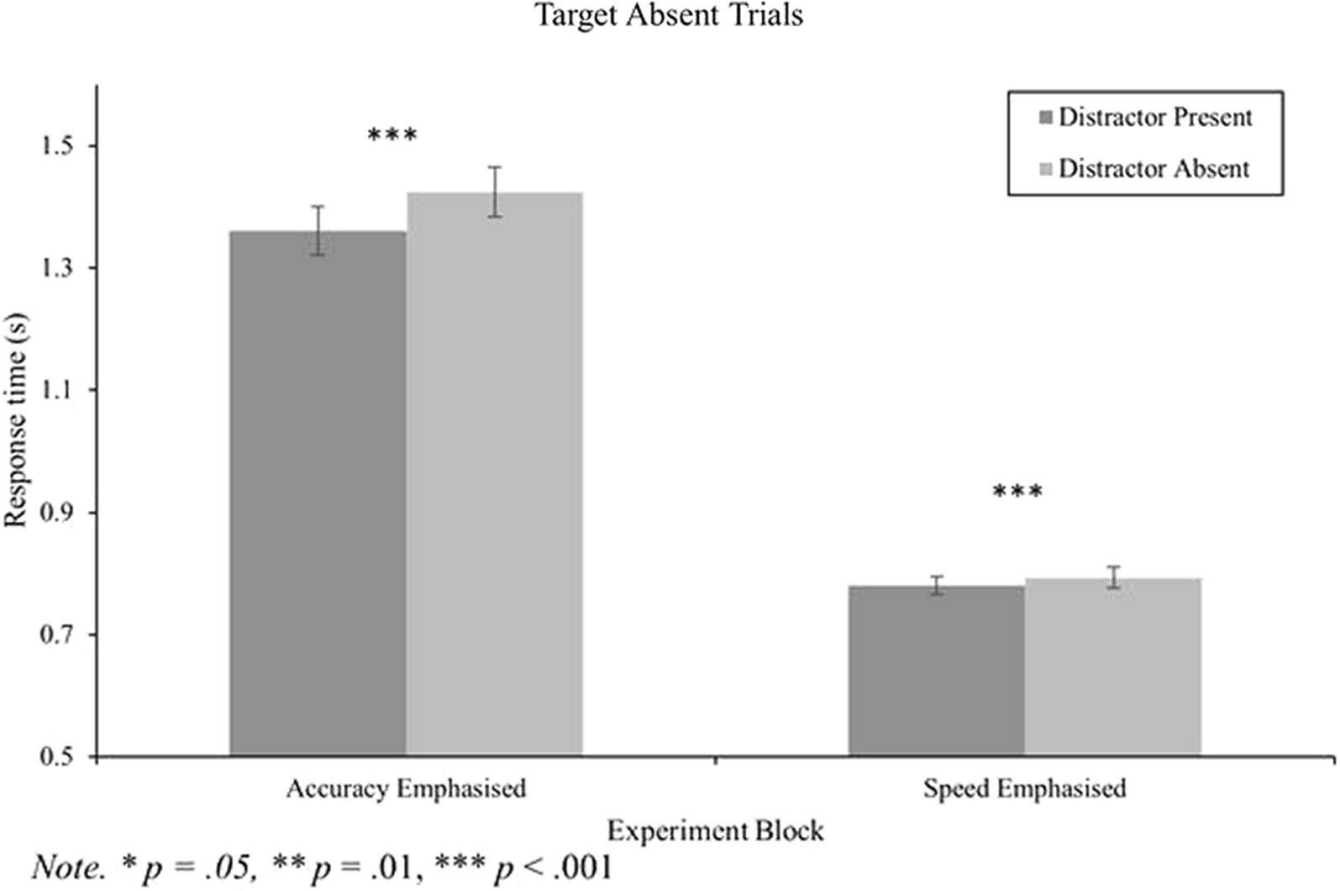
RT data for target absent trials as a function of distractor presence and experimental block. Within-subjects' error bars used (Cousineau, 2005)

A 2 (block: accuracy and speed) by 2 (distractor: present and absent) repeated measures ANOVA was conducted on the correct RT data for target absent trials. Overall, there was a large main effect of block, *F*(1, 165) = 392.54, *p* < 0.001, *η*_*p*_^2^ = 0.70, a main effect of distractor, *F*(1, 165) = 65.01, *p* < 0.001, *η*_*p*_^2^ = 0.28, and an interaction between distractor and block, *F*(1, 165) = 31.97, *p* < 0.001, *η*_*p*_^2^ = 0.16 (Fig. 2). To explore this interaction, we first conducted paired samples t-tests to check if the distractor speeding effect was present in both blocks. For the accuracy emphasis block, distractor present trials had significantly faster mean RTs compared to distractor absent trials, *t*(165) = 7.48, *p* < 0.001,* d* = 0.58 (Table 1). Likewise, for the speed emphasis block, mean RTs were faster in the distractor present condition compared to distractor absent condition, *t*(165) = 3.74, *p* < 0.001,* d* = 0.29 (Table 1). Together, these analyses suggest that speed versus accuracy instructions/feedback do not eliminate distractor-induced speeding for target absent search trials.

Next, we compared the magnitude of the distractor speeding effect across the two instruction/feedback conditions by computing ratios (distractor present RT/distractor absent RT) and conducting a paired samples t-test. A ratio was chosen as opposed to calculating difference scores because RTs were much faster in the speed emphasis block compared to the accuracy emphasis block (as expected and intended by the experimental manipulation). The mean ratio in the accuracy emphasis block was 0.9618 (*SD* = 0.0711). In contrast, the mean ratio for the speed emphasis block was 0.9878 (*SD* = 0.0736). Critically, the ratios for the speed and accuracy blocks statistically differed, *t*(165) = 3.39, *p* < 0.001,* d* = 0.26. Thus, it can be concluded that while distractor-induced speeding was present under both experimental conditions, the effect appeared to be smaller when response speeds were emphasized to participants.

#### Accuracy data for target present trials

**Fig. 3:**
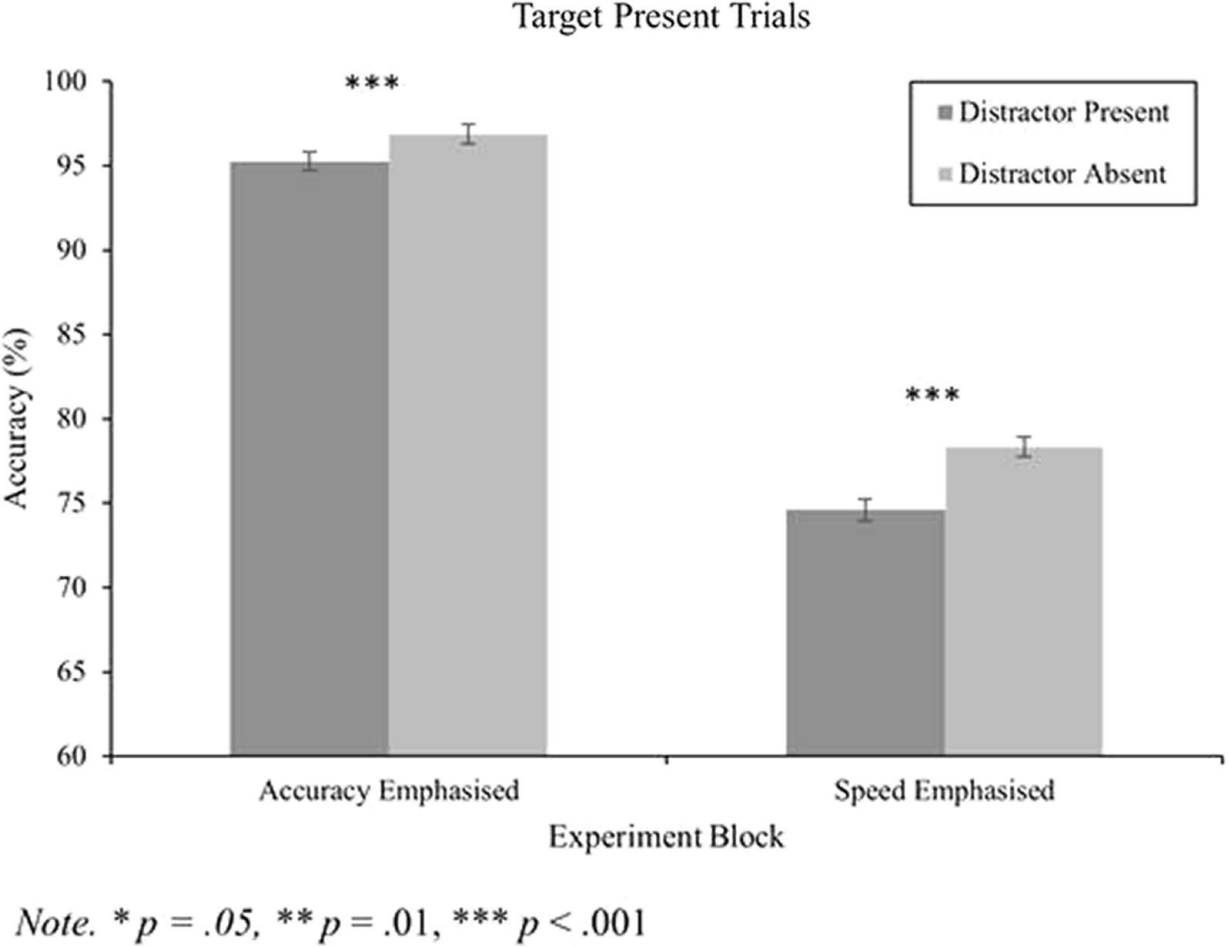
Accuracy data for target present trials as a function of distractor presence and experimental block. Within-subjects' error bars used (Cousineau, 2005)

A 2 (block) by 2 (distractor) repeated measures ANOVA was conducted on the accuracy data for the target present trials. Again, there was a large main effect of block, *F*(1, 165) = 320.36, *p* < 0.001, *η*_*p*_^2^ = 0.66, where accuracy was lower in the speed emphasis block compared to the accuracy emphasis block. There was also a main effect of distractor, *F*(1, 165) = 53.57, *p* < 0.001, *η*_*p*_^2^ = 0.25, and an interaction between block and distractor, *F*(1, 165) = 10.74, *p* = 0.001, *η*_*p*_^2^ = 0.06 (Fig. 3). Next, paired samples t-tests were used to see if the distractor-induced accuracy effect was present in both blocks. For the accuracy emphasis block, distractor present trials had significantly lower accuracy compared to distractor absent trials, *t*(165) = 5.27, *p* < 0.001,* d* = 0.41 (Table 2). Likewise, for the speed emphasis block, mean accuracy was lower in the distractor present condition compared to distractor absent condition, *t*(165) = 6.05, *p* < 0.001,* d* = 0.47 (Table 2).

Similar to the correct RT data for the target absent trials, we next explored potential differences in the magnitude of the distractor-induced accuracy effect by computing a ratio (distractor present accuracy/distractor absent accuracy) and comparing it for the speed and accuracy emphasis conditions using a paired samples t-test. The mean ratio in the speed emphasis block was 0.9530 (*SD* = 0.1141). The mean ratio in the accuracy emphasis block was 0.9836 (*SD* = 0.0417). The difference in the ratios was statistically significant, *t*(165) = 3.45, *p* < 0.001,* d* = 0.27. Thus, although neither speed nor accuracy instructions/feedback were successful in abolishing the distractor-induced decrease in target present accuracy, it was the case that compared to the speed emphasis block, emphasizing response accuracy did reduce the magnitude of target misses.

### Attentional capture

#### RT data for target present trials

**Fig. 4:**
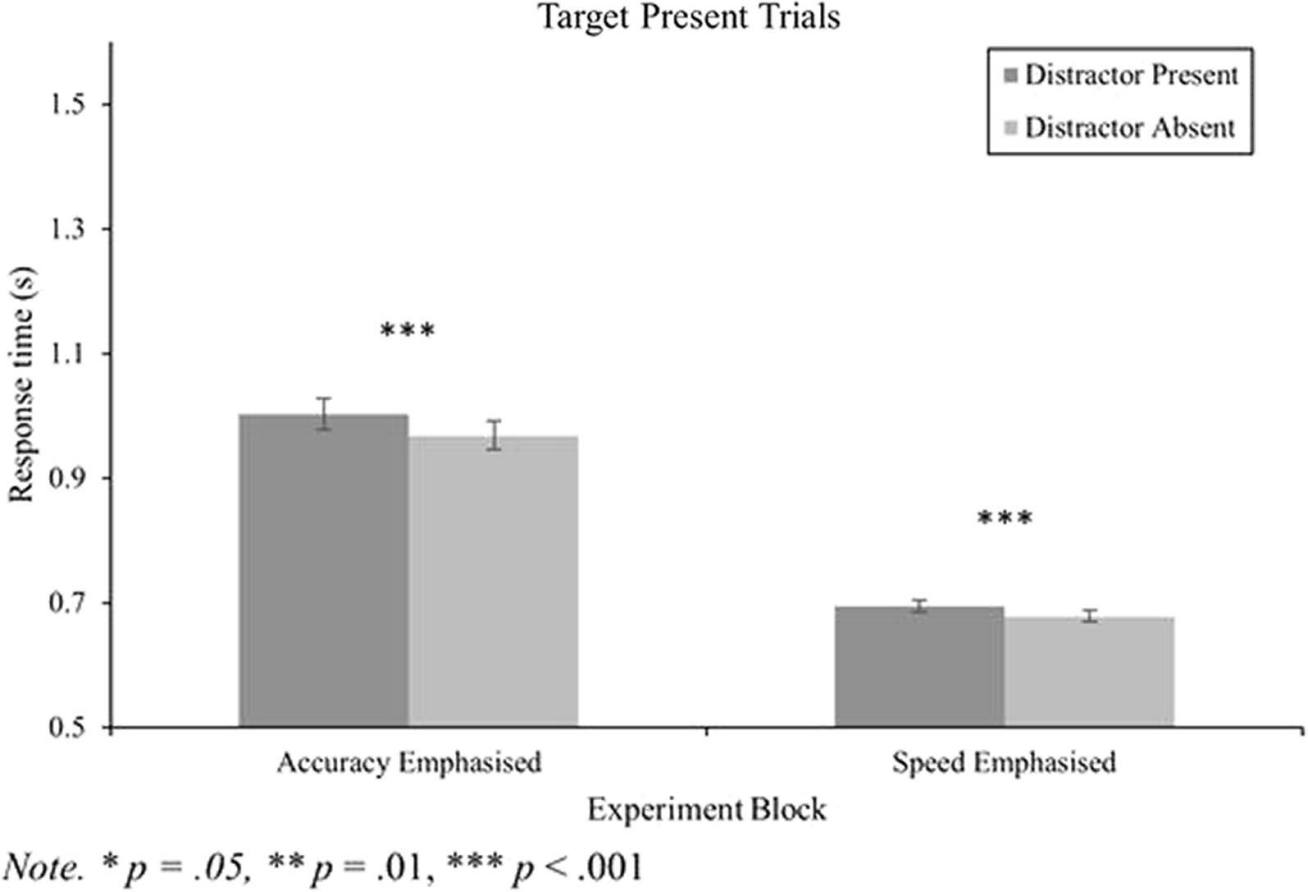
RT data for target present trials as a function of distractor presence and experimental block. Within-subjects' error bars used (Cousineau, 2005)

In addition to examining the distractor QTE, it is important to examine correct mean RTs in the target present condition to ensure that the distractor captured attention in the intended manner. As such, a 2 (block) by 2 (distractor) repeated measures ANOVA was conducted on the correct mean RT data for the target present trials. As shown in Fig. 4, there was a substantial main effect of block, *F*(1,165) = 302.60, *p* < 0.001, *η*_*p*_^2^ = 0.65, where response speeds were faster for the speed emphasis block compared to the accuracy emphasis block. There was also a main effect of distractor, *F*(1,165) = 36.74, *p* < 0.001, *η*_*p*_^2^ = 0.18, and an interaction between block and distractor, *F*(1,165) = 4.25, *p* = 0.041, *η*_*p*_^2^ = 0.03. Paired sample *t*-tests show that the distractor slowed search times for target present trials for both experimental blocks when it was present compared to absent [accuracy: *t*(165) = 4.35, *p* < 0.001,* d* = 0.34 and speed: *t*(165) = 5.15, *p* < 0.001,* d* = 0.40; Table 1], suggesting that regardless of instructions/feedback, the distractor captured attention.

To explore block differences in the magnitude of the attentional capture effect, ratios were calculated (i.e., distractor present RT/distractor absent RT) and compared using a paired samples t-test. The mean ratio for the speed emphasis block was 1.0253 (*SD* = 0.0645) whereas the mean ratio for the accuracy emphasis block was 1.0387 (*SD* = 0.0934). The difference in this ratio across conditions was not statistically significant, *t*(165) = 1.47, *p* = 0.143,* d* = 0.11, suggesting that the distractor captured attention similarly for both the speed and accuracy emphasis blocks.

#### Accuracy data for target absent trials

**Fig. 5:**
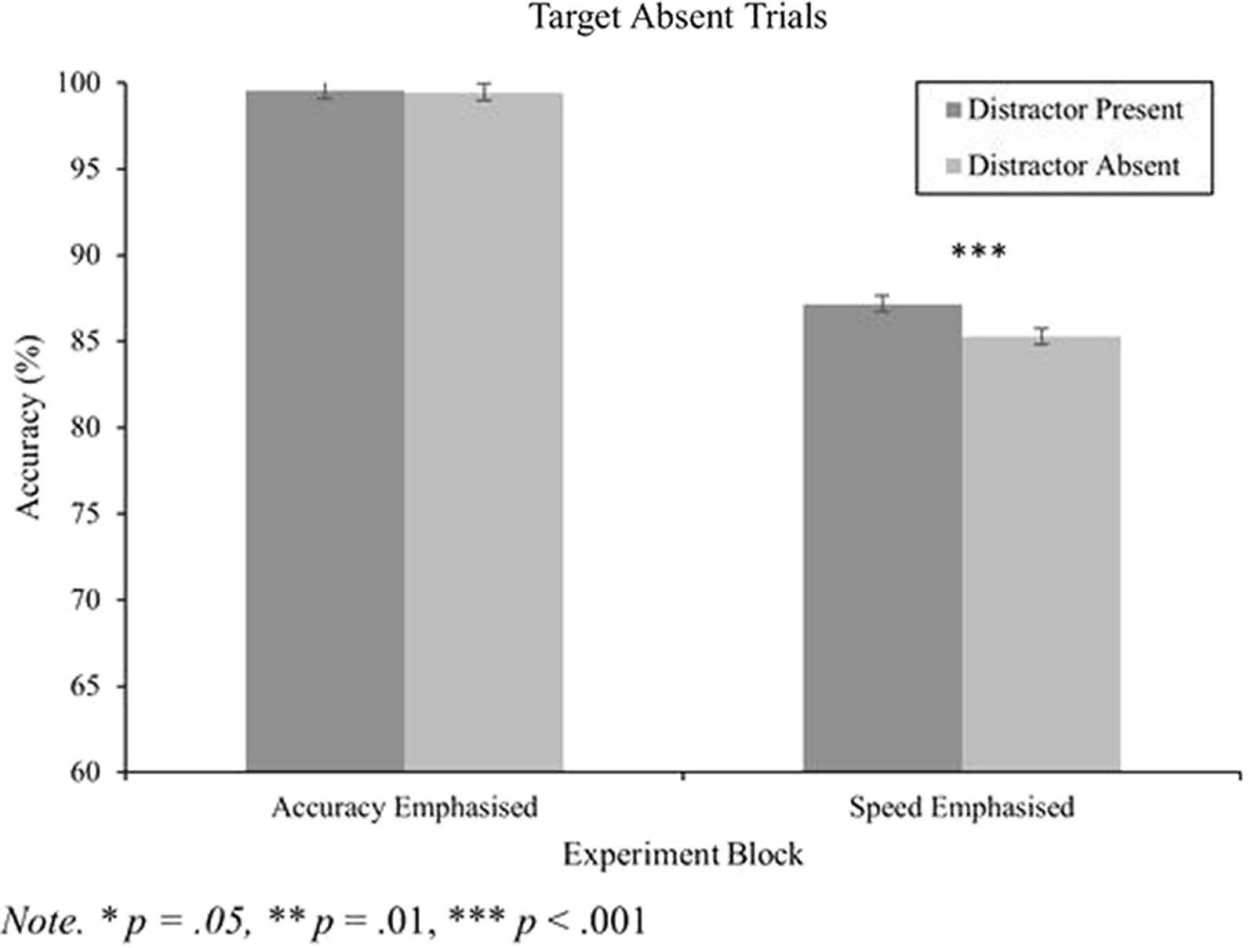
Accuracy data for target absent trials as a function of distractor presence and experimental block. Within-subjects' error bars used (Cousineau, 2005)

Finally, a 2 (block) by 2 (distractor) repeated measures ANOVA was conducted on accuracy data for the target absent trials. There was a large main effect of block, *F*(1, 165) = 215.99, *p* < 0.001, *η*_*p*_^2^ = 0.57, where accuracy was lower for the speed emphasis block compared to the accuracy emphasis block. There was a main effect of distractor, *F*(1,165) = 14.32, *p* < 0.001, *η*_*p*_^2^ = 0.08, and an interaction between block and distractor, *F*(1,165) = 11.55, *p* < 0.001, *η*_*p*_^2^ = 0.07. As shown in Fig. 5, the distractor had no effect on performance for the accuracy block [*t*(165) = 1.08 *p* = 0.280,* d* = 0.08 (Table 2)], where performance was already quite high. However, accuracy was higher when the distractor was present in the speed block [*t*(165) = 3.65, *p* < 0.001,* d* = 0.28 (Table 2)].

## Discussion

Given the potentially disastrous consequences of distractor-induced shifts in search strategies and quitting thresholds for some professional search contexts, the current study explored whether task instructions and trial-by-trial feedback may modulate the distractor QTE. Participants completed a search task across three blocks. One block provided no instructions or trial-by-trial feedback, whereas the remaining blocks encouraged fast or accurate responding. Overall, the speed and accuracy manipulations had very large effects on performance, where participants were faster in the speed emphasis block compared to the accuracy emphasis block, and more accurate in the accuracy emphasis block compared to the speed emphasis block (as indicated by the large main effects of experimental block in our analyses). Furthermore, although the QTE remained throughout the entire experiment, the size of the effect was modulated by task instructions and feedback. Specifically, the magnitude of the distractor-induced increase in target misses was smaller for the accuracy emphasis compared to the speed emphasis block. Despite this modulation occurring alongside an efficiency cost (search was much slower when accuracy rather than speed was encouraged), the results suggest that emphasizing response accuracy can lower the negative consequences of distractor-induced quitting.

That task instructions and feedback modulated the distractor QTE mirrors past research on quitting threshold effects more generally. Recall that task instructions encouraging fast or correct responding altered quitting thresholds in a visual search task that did not contain a salient distractor (McCarley, 2009). Furthermore, research suggests that the LPE can be reduced by including blocks of high target prevalence trials with feedback into an experiment with low target prevalence blocks and no feedback (Wolfe et al., 2007). However, as the current study combined instructions and feedback into a single task, it cannot be said with certainty if one, or both, of these factors drove changes in the distractor QTE. Given that trial-by-trial feedback may not be feasible in applied contexts such as baggage screening, it is, therefore, important for future research to test whether an instructions-only approach to reducing distractor-induced target misses is effective. Alternatively, based on the recent finding that instructions influence perceived target prevalence and thus affect quitting thresholds during visual search tasks (Cox et al., 2021), future research may wish to explore the influence of distractor expectations on the QTE.

Even if instructions and feedback do not completely remove the QTE, past research has found that the size of the salient distractor is crucial for observing the effect (Lawrence and Pratt, 2022). As such, one might question why task instructions are not as crucial. One possibility is that participants may not be consciously aware of a change in search strategy in the presence of a highly salient distractor and thus may not be especially vigilant to its effects on performance (even when they are told to perform the search task as accurately as possible). Therefore, it is important for future research to continue to explore the possible mechanisms driving the QTE to determine how to best alleviate its negative consequences.

Nonetheless, there is an alternative explanation for the data which must be considered. Specifically, it is possible that rather than changing the magnitude of the distractor QTE, the speed and accuracy instructions may have caused participants to perform close to a ceiling level performance, which, in turn, could have driven the interactions observed in the current study. However, this possibility seems unlikely as the metrics calculated to compare distractor effects across speed and accuracy emphasis blocks were ratios, which consider the overall differences in performance across blocks.^3^ Moreover, examining the data for the accuracy emphasis block shows that although accuracy is high overall in the task (as intended by the experimental manipulation), accuracy is most likely at ceiling for the target absent trials only (which is not a focus of the current study). Still, future research may wish to further explore this possibility by increasing the difficulty of the search task, which, in turn, would likely lower accuracy.

Apart from this alternative explanation, it is interesting to note that the target absent accuracy data was modulated by distractor presence in the speed emphasis condition. As shown in Fig. 5, there were a higher proportion of “target absent” responses for distractor present trials compared to distractor absent trials. One potential reason for this pattern of results is that in the speed emphasis block, the observer is under time pressure and must make a fast decision which could involve guessing. Critically, when the distractor is present, the observer has clear and salient information that at least one of the items in the search array is not the target. As such, this may make them more likely to have guessed a “target absent” response.

Furthermore, it is worth considering search performance in the baseline block compared to the speed and accuracy emphasis blocks. As expected, it appears that target misses were lower in the accuracy emphasis block compared to baseline (Table 2). However, response speeds in this block also appear numerically faster compared to those in the baseline block (Table 1). Although on the surface, these data could suggest that relative to baseline, participants enhanced response accuracy without much of a speed cost, this explanation appears unlikely. Instead, the speeding of RTs in the accuracy emphasis compared to the baseline block is likely due to practice effects, as participants always completed the baseline block first. Indeed, the baseline block was removed from statistical analyses because practice effects could have obfuscated any comparisons between the baseline block and the experimental conditions. On a related note, even though block order was randomized, in our analyses, 89 participants completed the accuracy emphasis block first whereas 77 completed the speed emphasis block first. Given that initial expectations can shape performance (e.g., Cox et al., 2021), an exploratory analysis including block order as a factor was conducted (see Additional file 1). This analysis suggested that the distractor-induced target absent speeding effect varied as a function of block and block order. As such, future research should systematically test the effects of expectations and practice on the distractor QTE explicitly (for a more detailed discussion, see supplementary material).

Finally, it is worth nothing that in task designs, where item locations are fixed instead of random, even filler items provide valuable information. For example, Little et al. (2015) demonstrated that detecting a single distractor (non-target) item provides sufficient information for a search for two targets to terminate when there are only two locations for targets to appear in. Howard et al. (2021) extended this logic even to detecting the absence of a stimulus in one location. In the standard, Moher (2020) paradigm used in the present study, the presence or absence of the salient distractor was uninformative about the target item, yet seemingly diverts attention away from the target-search process. It is plausible that the QTE may be altered if a predictive relationship between the distractor and target items was established such that processing either the target *or* distractor item provided task-relevant information. Furthermore, it is possible that the distractor present trials simply lowered the array of search items from 6 to 5, thus causing faster response times as there is one less plausible target item to inspect. However, this possibility seems unlikely given that the distractor slowed search times on target present trials, suggesting that it was indeed inspected. Nonetheless, future research may wish to further explore this possibility.

## Conclusion

The current study found that regardless of instructions and feedback, highly salient distractors lower quitting thresholds during target detection visual search. Nonetheless, it was the case that instructions and feedback emphasizing accuracy did lower the rate of target misses induced via distractor presence. Future research should, therefore, explore other potential mechanisms driving the QTE which could be manipulated alongside instructions/feedback to potentially minimize the effect.

### Supplementary Information

**Additional file 1**. Supplementary material.

## Data Availability

The data associated with this paper can be found on the Open Science Framework: https://osf.io/wn6u3/?view_only=8fd1200fb0a140c9bcf9dd6040e1e3f5 (Lawrence et al., 2023b). Research materials can be made available on request. The experiment was not pre-registered.

## Abbreviations

LPE: Low prevalence effect
QTE: Quitting threshold effect

## Footnotes

1 Mean RT was used as a response deadline as opposed to median RT due to programming limitations. A lower cutoff of 200 ms was used to calculate mean RT based on Moher (2020). An upper cutoff of 3 s was chosen pragmatically based on the logic that participants would not spend more than a few seconds searching to identify a target.

2 Despite outlier trimming, the assumption of normality was violated. However, given that ANOVA is robust to violations from normality (Blanca et al., 2017; Schmider et al., 2010), and because non-parametric analyses (signed ranks and Wilcoxon signed-rank tests) produced similar results for key QTE metrics compared to paired samples t-tests (i.e., distractor effects on target absent RTs and target present accuracy, as well as block differences in speeding and accuracy ratio metrics were similar), parametric analyses are reported here.

3 To further examine the interaction between block and distractor presence on target absent mean RTs, we applied a natural log transformation (1 + Ln(x)) to the data and re-ran the ANOVA and the paired samples t-tests. The analysis revealed a main effect of distractor, block, and an interaction (*p’s* < .05). For both the accuracy emphasis block, *t*(165) = 7.29, *p* < .001, *d* = .57, and the speed emphasis block, *t*(165) = 2.64, *p* = .009,* d* = .21, there was a significant effect of distractor presence.
