# Supplementary material for: Emphasizing responder speed or accuracy modulates but does not abolish the distractor-induced quitting effect in visual search
Source: Cogn Res Princ Implic. 2023 Oct 10;8:63. doi: 10.1186/s41235-023-00516-8 (PMC10564694; doi:10.1186/s41235-023-00516-8)
Supplement: Supplementary file 1 — Additional file 1. Supplementary material. [file 41235_2023_516_MOESM1_ESM.docx]

**Supplementary Material**

**Baseline Block Results**

To measure the distractor QTE in the baseline block, paired samples t-tests examining the effect of distractor presence on target absent RTs and target present accuracy were conducted. The analyses showed that target-absent response times were faster for distractor present (*M* = 1433ms, *SD* = 414ms) compared to distractor absent trials (*M* = 1493ms, *SD* = 436ms), *t*(165) = 6.23, *p* < .001, *d* = 0.48. Furthermore, target-present accuracy was lower when the distractor was present (*M* = 88.06%, *SD* = 9.40%) compared to absent (*M* = 91.31%, *SD* = 7.73%), *t*(165) = 6.60, *p* < .001, *d* = 0.51. Together, these findings indicate that a distractor QTE was present in the baseline block.

Paired samples t-tests were also used to examine the effect of the distractor on target present RTs and target absent accuracy data. Target-present RTs were slower for distractor present (*M* = 1127ms, *SD* = 279ms) compared to distractor absent trials (*M* = 1072ms, *SD* = 252ms), *t*(165) = 5.88, *p* < .001, *d* = 0.46. Furthermore, target-absent accuracy was similar when the distractor was present (*M* = 98.76%, *SD* = 2.45%) compared to absent (*M* = 99.02%, *SD* = 1.95%), *t*(165) = 1.31, *p* = .193, *d* = 0.10.

**Exploratory Analysis: Block Order Effects**

In the current study, the order of block completion was randomized for each participant. However, after data screening and cleaning, there were 89 participants who completed the accuracy block first and 77 who completed the speed block first. Given that initial expectations during visual search can shape performance (e.g., Cox et al., 2021), an additional exploratory analysis including block order as an effect was conducted for the target absent RT data and the target present accuracy data (the conditions most relevant to the QTE).

**RT Data for Target Absent Trials.** A 2 (block: accuracy, speed) by 2 (distractor: present, absent) by 2 (block order: speed first, accuracy first) ANOVA was conducted on the target absent correct mean RT data. This analysis revealed a main effect of block, *F*(1, 164) = 486.20, *p* < .001, η_p_^2^ = .75, a main effect of distractor, *F*(1, 164) = 63.51, *p* < .001, η_p_^2^ = .28, and a main effect of block order, *F*(1, 164) = 8.84, *p* = .003, η_p_^2^ = .05. Although there was no interaction between distractor and block order, *F*(1, 164) = 0.98, *p* = .323, η_p_^2^ = .01, there were significant interactions between block and block order, *F*(1, 164) = 51.20, *p* < .001, η_p_^2^ = .24, block and distractor, *F*(1, 164) = 30.76, *p* < .001, η_p_^2^ = .16, and critically, block, distractor, and block order, *F* (1, 164) = 5.19, *p* = .024, η_p_^2^ = .03.

To examine the three-way interaction between block, block order, and distractor presence, two separate ANOVAs exploring the relationship between block and distractor were conducted for the accuracy-first and speed-first participants separately. For the accuracy-first participants, there was a main effect of block, *F*(1, 88) = 366.05, *p* < .001, η_p_^2^ = .81, a main effect of distractor, *F*(1, 88) = 39.65, *p* < .001, η_p_^2^ = .31, and an interaction between block and distractor, *F*(1, 88) = 26.15, *p* < .001, η_p_^2^ = .23. Paired samples t-tests revealed the expected significant effect of distractor presence for the accuracy emphasis block, *t*(88) = 6.26, *p* < .001, *d* = 0.66 (*M _present_* = 1504ms, *SD _present_* = 463ms ; *M _absent_* = 1581ms, *SD _absent_* = 499ms). However, the effect of distractor presence for the speed emphasis block was non-significant, *t*(88) = 1.52, *p* = .133, *d* = 0.16 (*M _present_* = 755ms, *SD _present_* = 187ms ; *M _absent_* = 763ms, *SD _absent_* = 193ms).

For the speed-first participants, there was a main effect of block, *F*(1, 76) = 147.00, *p* < .001, η_p_^2^ = .66, a main effect of distractor, *F*(1, 76) = 25.39, *p* < .001, η_p_^2^ = .25, and an interaction between block and distractor, *F*(1, 76) = 7.15, *p* = .009, η_p_^2^ = .09. Paired samples t-tests revealed the expected significant effect of distractor presence for the accuracy emphasis block, *t*(76) = 4.22, *p* < .001, *d* = 0.48 (*M _present_* = 1195ms, *SD _present_* = 354ms ; *M _absent_* = 1243ms, *SD _absent_* = 386ms), as well as for the speed emphasis block, *t*(76) = 4.55, *p* < .001, *d* = 0.52 (*M _present_* = 810ms, *SD _present_* = 189ms ; *M _absent_* = 829ms, *SD _absent_* = 198ms). However, using the ratio metric (distractor-present RT/distractor-absent RT) for the distractor speeding effect, it was found that the ratio was similar for both blocks, *t*(76) = 1.17, *p* = .244, *d* = 0.13.

**Accuracy Data for Target Present Trials.** A 2 (block: accuracy, speed) by 2 (distractor: present, absent) by 2 (block order: speed first, accuracy first) ANOVA was conducted on the target present accuracy data. There was a main effect of block, *F*(1, 164) = 318.47, *p* < .001, η_p_^2^ = .66, and a main effect of distractor, *F*(1, 164) = 53.59, *p* < .001, η_p_^2^ = .25. There was no main effect of block order, *F*(1, 164) = 0.14, *p* = .711, η_p_^2^ < .01. Furthermore, although there was a significant interaction between distractor and block, *F*(1, 164) = 10.59, *p* = .001, η_p_^2^ = .06, no other interactions in the model were significant (*p*’s > .05).

**Summary.** The exploratory analysis suggests that while block order did not influence the relationship between distractor presence and experimental block on target present accuracy, it did influence the relationship between distractor presence and experimental block for the target absent correct mean RT data. Specifically, for participants who completed the accuracy block first, the distractor-based speeding for target absent trials emerged for the accuracy emphasis block, but not the speed emphasis block. However, for participants who completed the speed emphasis block first, the distractor based speeding effect was present in both the speed and accuracy emphasis blocks, and the magnitude of the effect was similar. Thus, consistent with previous research (e.g., Cox et al., 2021), it appears that initial expectations can shape quitting effects.

Perhaps the most striking finding of the exploratory analysis was that the distractor speeding effect was abolished for the participants who completed the accuracy block first when they completed the speed emphasis block. One possible reason for this finding are practice effects. When completing the initial accuracy emphasis block, one can assume that these participants had ample time to perform the task. Importantly, this time may have allowed the participants to gradually speed their responses as they became more practiced at the task without compromising their overall accuracy in the initial block. Consequently, by the time the participants were exposed to the speed emphasis block, they were already responding as quickly as possible, meaning that there was little room for distractor-based effects to be observed. Nonetheless, given that block order was randomized, and that order of completion was not of primary interest to the current study, it is important that future research systematically explore how practice effects and experience may shape the distractor induced quitting effect, especially in the context of instructions and feedback aimed at reducing the potentially negative consequences of the phenomenon.
